# Anticancer Potential of Fruit Extracts from* Vatica diospyroides* Symington Type SS and Their Effect on Program Cell Death of Cervical Cancer Cell Lines

**DOI:** 10.1155/2019/5491904

**Published:** 2019-04-17

**Authors:** Atchara Chothiphirat, Kesara Nittayaboon, Kanyanatt Kanokwiroon, Theera Srisawat, Raphatphorn Navakanitworakul

**Affiliations:** ^1^Department of Biomedical Sciences, Faculty of Medicine, Prince of Songkla University, Hat Yai, Songkhla 90110, Thailand; ^2^Department of Agricultural Science and Technology, Faculty of Science and Industrial Technology, Prince of Songkla University, Surat Thani Campus, Surat Thani 84000, Thailand

## Abstract

*Vatica diospyroides *Symington is locally known as Chan-Ka-Pho in Thailand. Ancient people have used it as therapeutic plant for cardiac and blood tonic cure. The purpose of this study was to investigate the potential cytotoxicity and selectivity of the extracts from* V. diospyroides* type SS fruit on cervical cancer HeLa and SiHa cell lines and to examine its underlying mechanism of action. MTT assay revealed that the extracts showed inhibition of cell survival in a dose-dependent manner and exhibited highly cytotoxic activity against both HeLa and SiHa cells with IC_50_ value less than 20 *μ*g/mL along with less toxicity against L929 cells. Acetone cotyledon extract (ACE) showed the best selectivity index value of 4.47 (HeLa) and 3.51 (SiHa). Distinctive morphological changes were observed in ACE-treated cervical cancer cells contributing to apoptosis action. Flow cytometry analysis with Annexin V-FITC and PI staining precisely indicated that ACE induced apoptosis in HeLa and SiHa cell lines in a dose-dependent manner. Treatment of ACE with half IC_50_ caused DNA fragmentation and also activated increasing of bax and cleaved caspase-8 protein in HeLa cells after 48 h exposure. The results suggest that ACE has potent and selective cytotoxic effect against cervical cancer cells and the potential to induce bax and caspase-8-dependent apoptosis. Hence, the ACE could be further exploited as a potential lead in cancer treatment.

## 1. Introduction

Cervical cancer is the fourth most commonly diagnosed cancer in females worldwide, with 528,000 new cases and 266,000 deaths annually [1]. The incidence and mortality rates of cervical cancer have considerably reduced in developed countries through the application of cervical cancer screening tests for high-risk human papillomavirus and through Papanicolaou smear; however, the incidence rate of this disease remains high (i.e., 80%) in developing countries, where 70% of the patients are at the advanced stage of the disease, and the age at diagnosis of the patients is slightly decreasing [2, 3]. The recurrence rate of cervical cancer is still high in developing countries, and radical surgery affects the long-term recovery and survival rate of patients after curative resection [4]. Moreover, radioactive rays and most anticancer drugs suppress DNA duplication or damage DNA in order to kill cancer cells divided rapidly. Meanwhile, they also affect normal cells to cause adverse side effects, such as bone marrow function inhibition, nausea, vomiting, and alopecia [5, 6]. Therefore, new forms of therapy should be discovered to improve the clinical outcome of patients with cervical cancer.

Natural crude extracts and biologically active compound isolated from plant have been widely used in traditional Thai medicine for treatment of various cancers. Interestingly,* Vatica diospyroides* Symington (VDS) including two distinct types of tree, namely, LS and SS forms, the species most interested and reported by our laboratory, is the medicinal plant known in Thai as Chan-Ka-Pho, which is a valuable source of human cancer chemopreventives, such as phytochemical constituents like saponins, cardiac glycosides, flavonoids, tannin and terpenoids, and resveratrol tetramers [7–10]. The resveratrol derivatives, namely, Vaticaphenol and Vaticanol, have been found to play an important role in cardiovascular treatment and to be cytotoxic against various human cancer cell lines such as human oral epidermoid (KB), colon cancer (Col2), and breast cancer (BC1) cell lines [8, 9]. Srisawat and colleagues reported that acetone and methanol extracts of VDS type LS contain saponins, anthraquinones, and terpenoids which have various biological activities including antioxidant, anti-inflammatory, and anticancer activities. Moreover, these extracts have been shown to be highly cytotoxic against MDA-MB-468 and to have less toxicity in normal Vero cells [7]. In addition, the cotyledon and pericarp extracts of VDS type SS have been reported as antiproliferative properties and induced apoptosis in MDA-MB-231, MDA-MB-468, and MCF-7 breast cancer cells [11, 12]. Although this species is reported as an efficient medicinal plant, the cytotoxicity against cervical cancer and response of cell lines to the plant extract have never been described. The aim of present study was to determine the antiproliferative effects of VDS type SS fruit extracts through the induction of apoptosis in human cervical cancer cell lines including HeLa and SiHa.

## 2. Materials and Methods

### 2.1. Cell Cultures

Human cervical cancer cell lines used in this research are HeLa (ATCC® CCL-2™) and SiHa (ATCC® HTB-35™). These cell lines were purchased from the American Type Culture Collection (ATCC) (Manassas, VA). L929 fibroblast cell line was kindly provided from Assoc. Prof. Dr. Jasadee Kaewsrichan, Department of Pharmaceutical Chemistry, Faculty of Pharmaceutical Sciences, Prince of Songkla University (Songkhla, Thailand). These cell lines were cultured in Dulbecco's modified Eagle's medium (DMEM) supplemented with 10% fetal bovine serum (FBS), L-glutamine, streptomycin (100 *μ*g/mL), and penicillin (100 U/mL). All cells were incubated in humidified air with 5% CO_2_ incubator at 37°C.

### 2.2. Preparation of VDS Extract

10-year-old fruit of* V. diospyroides* type SS was collected in collector number T Srisawat 002 and the preparation of acetone and methanolic extracts from the fruit including cotyledon and pericarp was described by a previous method [7]. Briefly, the fruit cotyledon and pericarp were separately cut to small pieces and completely air-dried in shadow. Dried pieces of each sample were separately extracted with acetone or methanol for 5 days in agitated condition. The solvent extract was then transferred to a new container, filtered through a cotton fabric. The extracts were evaporated at room temperature using a rotor evaporator (Heidolph Rotary Evaporator, D-91126, Germany) under reduced pressure to dry residue and stored in cool and dark condition. Then, extracts were dissolved with dimethyl sulfoxide (DMSO) with a final concentration of DMSO in extracts less than 0.5%. Extracts of fruit pericarp and cotyledon were kept separately and stored in darkness at −20°C prior to test against cervical cancer (HeLa and SiHa) and normal fibroblast (L929) cell lines in a cytotoxicity test.

### 2.3. Cytotoxicity and Selectivity Assay

The cytotoxicity effects of acetone or methanolic extract of pericarp and cotyledon against cervical cancer cell lines were determined by MTT (3-(4,5-dimethylthiazol-2-yl)-2,5-diphenyltetrazolium bromide) dye uptake assay. The HeLa and SiHa cell lines were seeded in 96-well plates containing 5 × 10^3^ cells in 150 *μ*L of completed medium per well. Cells were permitted to adhere for 16 h and then treated with the extracts diluted to 5-80 *μ*g/mL concentration in a medium. In addition, DMSO alone was added to another set of cells as a control. The cells were incubated for 72 h and then washed with phosphate buffer saline (PBS) prior to the addition of 100 *μ*L of 0.5 mg/mL MTT solution into each well. The formazan crystals were dissolved with DMSO (150 *μ*L) before the absorbance at 570 nm and 650 nm was measured on SpectraMax M5 multimode microplate reader (Molecular Devices, USA). The whole experiment was replicated in three independent times, and cell viability determination and fitting of response curves followed the previously described method [11]. The 50% inhibitory concentration (IC_50_) of the crude extracts was calculated from fitted response curves and determined according to the US National Center Institute and Geran et al. [13] and labeled for the activity levels as follows: IC_50_ ≤ 20 *μ*g/mL = highly active, IC_50_ 21-200 *μ*g/mL = moderately active, IC_50_ 201-500 *μ*g/mL = weakly active, and IC_50_ > 500 *μ*g/mL = inactive.

The selectivity index (SI) was determined by the ratio of the IC_50_ value of the extracts on normal cells (L929) to the IC_50_ value of the extracts on cancer cells (HeLa or SiHa). Samples with an SI greater than 3 were considered to have a high selectivity towards cancer cells [14, 15].

### 2.4. Apoptosis Investigation by Inverted Microscopy and Flow Cytometry

Cells were seeded in 6-well plates at density of 1.5 × 10^5^ cells per well for 16 h prior to treatment with the acetone cotyledon extracts (ACE) at half IC_50_, IC_50_, and 2-fold IC_50_ concentration for 48 h. The mode of cell death was observed by Olympus Culture Microscope model CK40 with 100x magnification (Olympus Corporation, Tokyo, Japan) and determined by Annexin V-FITC/Propidium iodide (PI) binding assay following manufacturer's protocol (BD Pharmingen™, USA). Briefly, control and treated cells were washed with PBS and harvested by adding of 300 *μ*L of trypsin-EDTA. The harvested cells were resuspended in 1x binding buffer (0.1 M Hepes, 0.1 M NaOH pH 7.4, 1.4 M NaCl, 25 mM CaCl_2_) at a concentration of 1 × 10^6^ cells/mL. 5 *μ*L of Annexin V-FITC and PI was subsequently added to the suspensions. The suspensions were vortexed gently, and 400 *μ*L of 1x binding buffer was added and incubated at room temperature for 15 min in darkness. The flow cytometer was a fluorescence activated cell sorter (FACS) Calibur (Becton Dickinson Biosciences [BDB], San Jose, CA), equipped with a 488 nm argon ion laser. A total of 10,000 events were acquired with CellQuest software. The populations of viable, early apoptotic, late apoptotic, and dead cells in each experiment were analyzed, and dot plot diagrams were generated with WinMDI version 2.9 software (Scripps Institute, La Jolla, CA).

### 2.5. DNA Fragmentation

HeLa cells at 10^7^ cells/ml were seeded in 100 mm culture vessel. After overnight incubation, medium was replaced, and fresh medium was added along with half IC_50_ doses of ACE and subsequently incubated for 0.5, 24, 48, 72, 96, and 120 h. Triton-X 0.01% was used as a positive control of DNA fragment pattern. After treatment, cells were harvested and lysed with lysis buffer containing 50 mM Tris-HCl, pH 8.0, 20 mM EDTA, pH 8.0, 5% and triton X-100 for 20 min on ice. The total genomic DNA was then isolated with phenol/chloroform/isoamyl alcohol (25:24:1) and treated with RNase (20 *μ*g/mL) at 37°C for 30 min. DNA was precipitated with cold isopropanol and washed with 75% cold ethanol. DNA was electrophoresed in 2.5% agarose gel and subsequently visualized using ethidium bromide under ultraviolet transilluminator and photograph in gel documentation system (Bio-Rad, CA, USA)

### 2.6. Western Blot Analysis

HeLa cells were treated with the ACE at half IC_50_ for 0, 24, 48, and 72 h. The cells were trypsinized and harvested by centrifugation. Cell pellets were washed once in cold PBS and lysed in RIPA buffer (Pierce Biotechnology, IL, USA). Protein concentration was measured by Bradford assay (Bio-Rad, CA, USA). Protein sample with 40 *μ*g was subjected to electrophoresis using 12% SDS-PAGE in running buffer at constant 120 V for 2 h and electrotransferred onto a PVDF (polyvinylidene difluoride) membrane. The membranes were blocked in 5% non-fat milk in Tris-buffered saline with 0.1% (v/v) Tween-20 (TBS-T) for 1 h at room temperature. After blocking, the membranes were then washed twice with TBS-T for 10 min. Each membrane was incubated overnight at 4°C, shaking continuously primary antibodies (1: 1,000; diluted with 1% non-fat milk in TBS-T) specific with bax, caspase-8, cleaved caspase-8, bcl-2, and *β*-actin used as the internal control. After incubation, the membranes were washed with TBS-T for three times (10 min/time) and incubated with secondary antibody (anti-rabbit IgG horseradish peroxidase) at 1: 5,000 in 1% non-fat milk in TBS-T for 2 hours. Then, membranes were washed in TBS-T for three times and the last washing was using TBS for 10 min. Finally, the protein expressions were detected by a chemiluminescent detection kit (Pierce, IL, USA).

### 2.7. Statistical Analysis

The experiments were repeated in three times. Data were expressed as means ± standard deviation (SD). One-way ANOVA was used to compare means. A p value < 0.05 was considered to indicate a statistically significant difference.

## 3. Results

### 3.1. Do VDS Extracts Induce Potent Cytotoxic Effects in Cervical Cancer Cells and Normal Fibroblast Cells?

**Figure 1:**
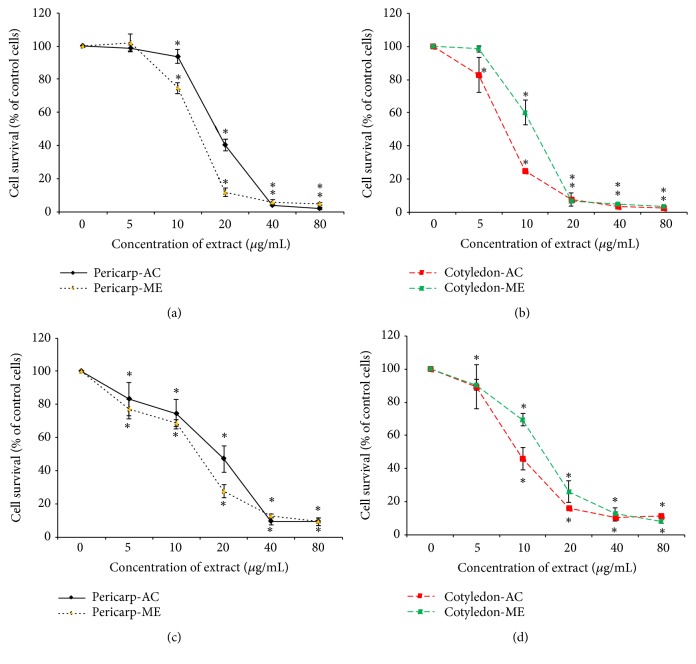
Cell survival curves on HeLa (a-b) and SiHa (c-d) cell lines are determined by MTT assay for 72 h of treatment with VDS fruit type SS crude extracts with concentrations of 0, 5, 10, 20, 40, and 80 *μ*g/mL (ME, methanol, and AC, acetone). Data are shown as mean ± SD of three independent experiments (*∗p* < 0.05; the mean difference is significant at the 0.05 level compared to control by one-way ANOVA).

The antiproliferative activity of cotyledon and pericarp extracts of VDS type SS against cervical cancer cells and normal fibroblast cells was evaluated by MTT assay. Different concentrations of the extracts (0-80 *μ*g/mL) were tested against the cancer cells and normal cells. Both HeLa and SiHa cells showed substantial dose-dependent susceptibility to the treatment of different concentrations of the extract as shown in Figure 1. The number of viable cancer cells exposed to the extract treatment was reduced significantly as the dose increased. The IC_50_ value of the extracts after 72 h intervals was estimated from fitted response curves and shown in Table 1. The ACE exhibited both the strongest inhibition and the highest selectivity in both HeLa and SiHa cells with IC_50_ of 7.69 ± 0.44 and 9.81 ± 1.38 *μ*g/mL, respectively. In addition, the SI values of ACE in HeLa and SiHa cells compared with L929 cells were 4.47 and 3.51, respectively (Table 2). On the other hand, other extracts showed lower SI values for both cervical cancer cell lines. Our results indicated that the ACE has potent cytotoxic activity and a good selectivity against cervical cancer cells. Therefore, we only selected this extract for further experiments.

### 3.2. Effect of ACE on the Cell Morphology

**Figure 2:**
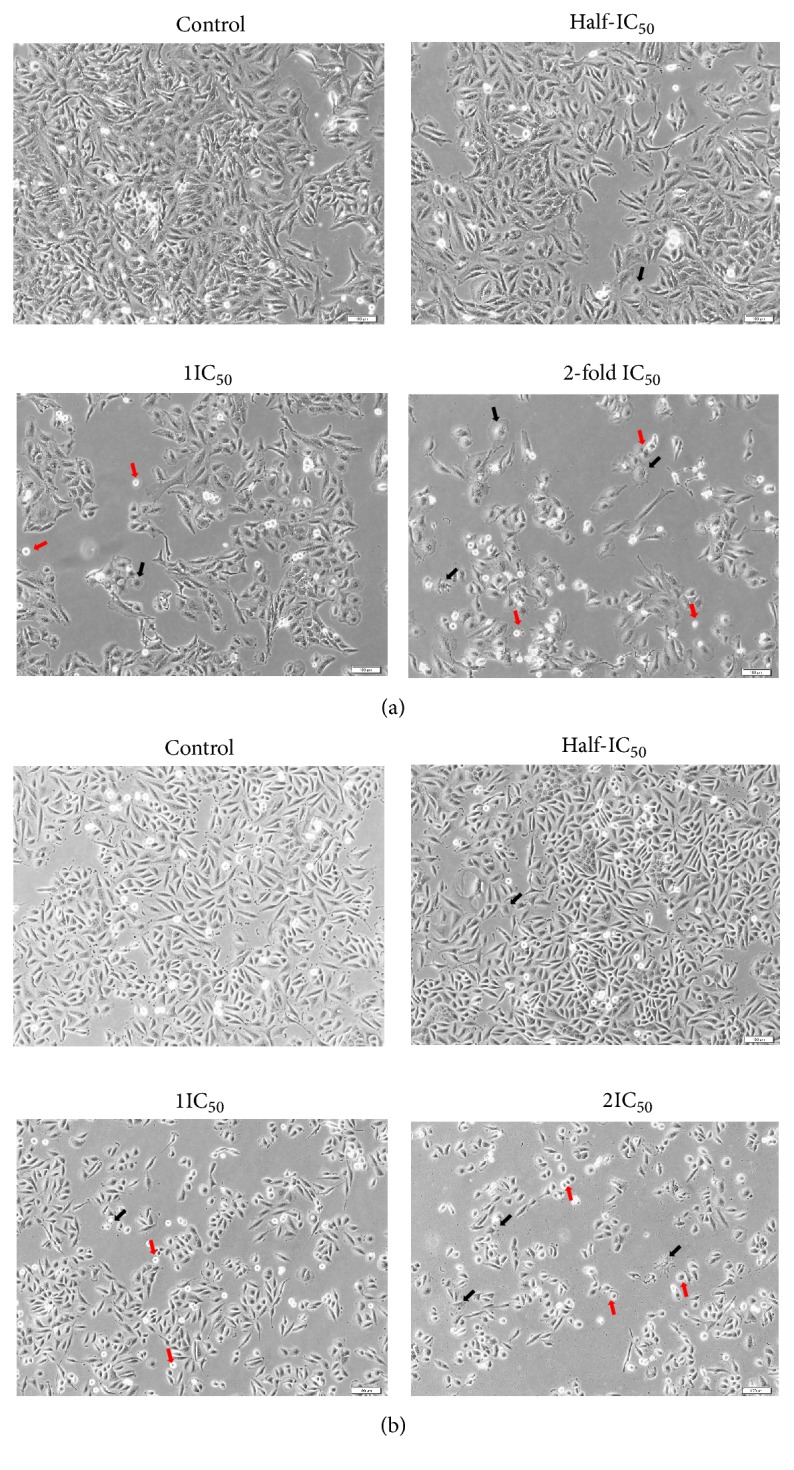
Morphological changes of (a) HeLa and (b) SiHa cells treated with ACE at half IC_50_, IC_50_, and 2-fold IC_50_ for 48 h. The cells were evaluated under an Olympus inverted microscope using 10X objective lens (magnification 100X). Morphology of cell changes in star-shape and cell shrinkage were indicated by black and red arrows, respectively. Scale bar: 10 *μ*m.

From the cytotoxic results, it was obvious that ACE was very effective against HeLa and SiHa cells with lowest IC_50_ value. Morphology changes of HeLa and SiHa cells were observed in which both cells were treated with half IC_50_, IC_50_, and 2-fold IC_50_ of ACE for 48 h. Compared with the untreated cells, the majority of the ACE-treated HeLa and SiHa cells changed from spindle to star-shaped, some of which become damaged and shrink morphology (Figure 2). Meanwhile, their growth was inhibited in a concentration-dependent manner. These morphological changes are typical of apoptosis.

### 3.3. ACE Induces Apoptosis in HeLa and SiHa Cells

**Figure 3:**
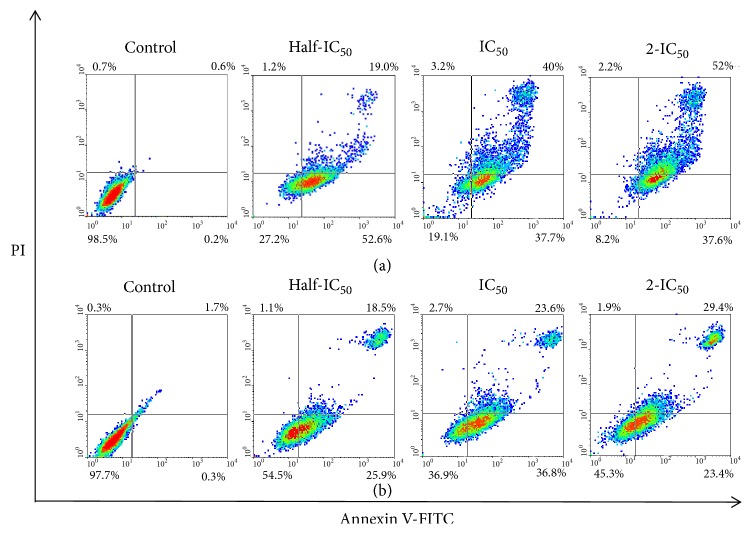
Apoptosis induction effect of cotyledon-acetone extract (ACE) at half IC_50_, IC_50_, and 2-fold IC_50_ on HeLa (a) and SiHa (b) cells for 48 hours determined by Annexin V-FITC/PI flow cytometry. Dot plot diagrams show percentage of cell populations divided to viable (lower left quadrant; Annexin V and PI negative), early apoptotic (lower right quadrant: Annexin V positive and PI negative), late apoptotic (Upper right quadrant; Annexin V and PI positive), and dead cells (Annexin V negative and PI positive).

Based on morphology observation, we therefore elucidated whether ACE induces apoptotic or necrotic cell death using flow cytometry. Double staining by Annexin V-FITC and PI was performed to examine the apoptotic potential of the extract. After HeLa and SiHa cells were treated with different concentrations of ACE for 48 h, cells were stained with Annexin V-FITC and PI, which can distinguish between viable, early apoptotic, late apoptotic, and dead cell populations as presented in the lower left, lower right, upper right, and upper left of the quadrant of the density plot, respectively. Compared to untreated cells, we found that ACE treatment at half IC_50_ reduced the viable HeLa cell population to 27.2% and increased the percentage of early apoptotic cells to 52.6%. Interestingly, the populations of viable and early apoptotic cells were dramatically decreased, while the population of late apoptosis was significantly increased from 19 to 52% with increasing dose of ACE. This result indicated that apoptosis occurred in HeLa cells by the extract in a dose-dependent manner (Figure 3(a)). On the other hand, treatment with the ACE at half IC_50_ separated the SiHa cells to viable, early apoptotic, late apoptotic, and dead cell populations (54.5, 25.9, 18.5, and 1.1%) as shown in Figure 3(b). Notably, at IC_50_ dose level, the population of viable cell was reduced to 36.9% and that of early apoptotic cells was increased to 36.8%. Additionally, the numbers of viable and early apoptotic cells were decreased with higher dose (2-fold IC_50_) of ACE. Only population of late apoptotic cells was increased with increasing dose, indicating that induction of apoptosis mode occurred in SiHa cells but there was no relevance to dose response.

**Figure 4:**
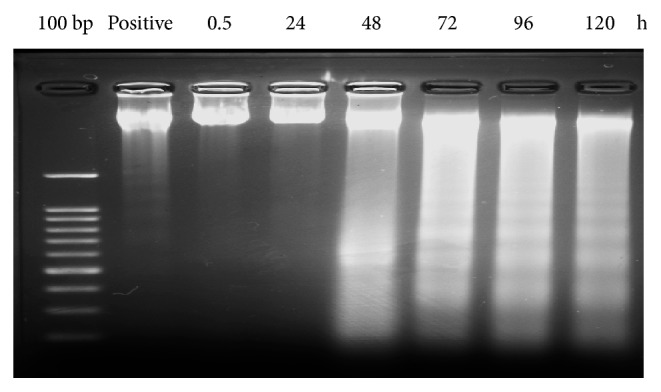
DNA fragmentation pattern in HeLa cells treated with ACE at half IC_50_ by 2.5% agarose gel electrophoresis and ethidium bromide staining. Lane 1: 100 bp DNA ladder, lane 2: positive control (0.01% triton-x 100), lanes 3-8: ACE-treated cells for 0.5, 24, 48, 72, 96, and 120 h, respectively. The data are representative of three independent experiments addressed under the same conditions.

We determined DNA fragmentation in order to clarify whether the action of the ACE was associated with apoptosis or not. As shown in Table 1 and Figure 1, the ACE was strongly effective on both cervical cancer cell lines; only HeLa cells were then used to represent the event. In order to avoid necrotic cell death from acute toxicity with high extract dose levels, we incubated the cells with the extract using half IC_50_ concentration. DNA fragmentation was found on HeLa cells at 48, 72, 96, and 120 h after exposure (Figure 4). Therefore, the ladder fragmentations of DNA and characteristic morphological changes indicated that the cytotoxic effect of the extract was mediated through the induction of apoptosis.

### 3.4. Apoptosis Mode by Protein-Based Analysis

The Western blot analysis was used to assess the underlying mechanism for the induction of apoptosis. We used actin protein as an internal control. Bax and cleaved caspase-8 were upregulated, whereas bcl-2 and the inactive form of caspase-8 were downregulated at their half IC_50_ at 48 h after treatment. These data showed that bax, bcl-2, cleaved caspase-8, and caspase-8 were involved in the apoptosis of HeLa cells induced by the extract.

## 4. Discussion

A traditional medicine has been attended as one of the valuable sources of chemopreventive drugs that could reduce morbidity as well as side effects resulting from conventional chemotherapy. Previous studies have shown that the crude extracts of VDS type LS and SS fruit have strongly appreciable cytotoxicity in breast cancer cell lines underlying apoptosis mechanism [7, 11, 12, 16]. In the present study, we evaluated the anticancer properties of the crude extracts of VDS type SS in various fruit parts and organic solvents for extraction on HeLa and SiHa human cervical cancer cells along with demonstrating its mode of action by apoptosis. Our results exhibited that acetone and methanol extracts of cotyledon and pericarp from VDS significantly inhibited the growth of both cancer cell lines with IC_50_ < 20 *μ*g/mL. The criteria of American Cancer Institute considered a crude extract promising for further development of a potential anticancer natural compound with a higher cytotoxic activity based on the IC_50_ values lower than 30 *μ*g/mL [17]. Based on these criteria, these crude extracts from VDS showed a potential that may be developed as a new anticancer drug. Moreover, the data from MTT assays showed that VDS extracts could inhibit growth of HeLa and SiHa cells in a dose-dependent manner (Figure 1). Notably, ACE exhibited greater cytotoxic effect than other extracts in HeLa and SiHa cells with IC_50_ at 7.69 ± 0.44 and 9.81 ± 1.38 *μ*g/mL, respectively. Nonetheless, these extracts possessed less cytotoxic effect on normal L929 fibroblast cells (Table 1). Our results are consistent with previous studies showing that ACE from VDS type SS fruit exhibited cytotoxicity to MCF-7 and MDA-MB-231 human breast cancer with IC_50_ at 16.21 ± 0.13 and 30 ± 4.30 *μ*g/mL, respectively. Our study highlights the cytotoxic activity of ACE against different cancer types, and cervical cancer may be more sensitive to the extract than breast cancer cells. Moreover, there are some possible reasons supporting the different toxicity effect of the four extracts of VDS based on the phytochemical reports on the type LS fruit carried out by Srisawat et al. [7]. Both acetone and methanol have been widely used for extraction in order to recover terpenoids, anthraquinones, and saponins which mainly have been known as chemopreventive agents for various cancer therapies [18–20]. Moreover, the resveratrol tetramers obtained from the stem of VDS have been proven to possess therapeutic properties against various cancers [8]. In addition, one of the reasons is due to difference between the levels of those compounds in the crudes. Therefore, we assumed that the antiproliferation effect of VDS type SS against HeLa and SiHa cells might be caused by the phytochemicals and such natural compounds. Its level may have significant role in ACE towards the cytotoxicity on both cancer cells. The selectivity index (SI) generally indicated the safety of an extract used for anticancer therapy. The index was calculated based on the ratio of the IC_50_ value of the extracts on normal L929 cells to human cancer cells (HeLa and SiHa). SI value greater than 3 was regarded as highly selective [14, 15]. Among these extracts, the ACE displayed high selectivity with a SI value of 4.47 and 3.51 for HeLa and SiHa cells, respectively (Table 2). In accordance with study of Berrington and Lall [21], acetone extracts of* Origanum vulgare* (oregano) and* Laurus nobilis* (bay leaf) have strongly inhibited the proliferation of the HeLa cells. High selectivity in cytotoxic response between cancer and normal cell lines enhances prospect of this extract to contain important compound which could serve as a novel anticancer drug.

Subsequently, the mode of cell death induced by the ACE at half IC_50_, IC_50_, and 2-fold IC_50_ was further explored. In view of the morphological features of the cells observed in response to ACE using inverted microscopy, the ACE induced potent morphological changes in cervical cells including cell shrinkage and star-shape with membrane blebbing which represent a typical apoptosis. Most anticancer agents exert cytotoxic effects on malignant cells by inducing apoptosis [22]. Induction of apoptosis by the ACE was further examined by flow cytometry. Apoptosis is demonstrated by translocation of phospholipid phosphatidylserine (PS) from the inner to outer layer of plasma membrane for phagocyte recognition during early stage of apoptosis [23]. Therefore, the externalization of PS to the outer layer is detectable by Annexin V-FITC for identification of early stage apoptosis. Concurrently, PI is nucleic acid binding red fluorescent dye which is impermeable to live cells and early apoptotic cells, but it can penetrate to the nucleus and stain the DNA of late apoptotic and necrotic cells when the cytoplasmic membrane integrity is lost [24–26]. Treatment with ACE revealed that the ACE induced the exposure of PS on the surface of HeLa in a dose-dependent manner. While in SiHa the treatment with half IC_50_ and IC_50_ of the ACE resulted in more percentage of early and late apoptotic cells, the treatment with high dose (2-fold IC_50_) resulted in less cells present in the early apoptosis stage than late apoptosis. To explain the differences in late apoptosis in HeLa and SiHa cells treated with VDS, it is important to consider that late apoptosis is irreversible, and each process could differ depending on the genetic background of the cells including their human papillomavirus (HPV) status and the viral copy number. For example, HeLa (adenocarcinoma) has approximately 10-50 integrated copies of HPV18, whereas SiHa (squamous cell carcinoma) contains 1-2 integrated copies of HPV 16 genome [27, 28]. Moreover, the rate of replication is also different in both cell types. [29]. Interestingly, we noticed degradation of DNA in terms of fragmentation at 200-1000 bp when HeLa cells were treated with ACE at half IC_50_ after 48-120 h exposure. In addition, other studies have shown that betulinic acid from* Melaleuca cajuputi *caused DNA ladder in 100-1500 bp fragment in HL-60 cells [30]. Therefore, DNA ladder formation indicated that the cytotoxic effect of ACE caused inhibition in the growth of cervical cancer cells through apoptosis, and this event had not recovered.

**Figure 5:**
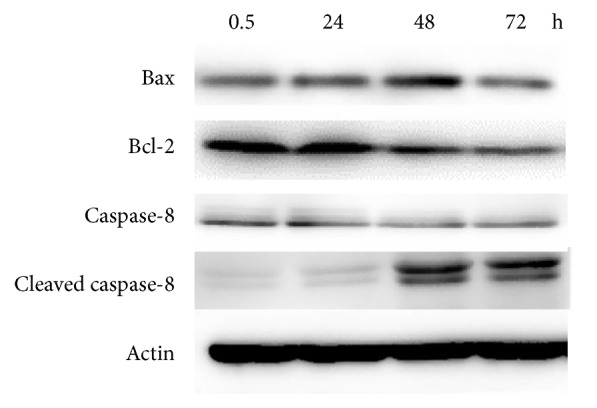
Apoptosis induction effect of ACE at half-IC_50_ on protein expression of bax, bcl-2, caspase-8, and cleaved caspase-8 in HeLa cells using Western blot analysis. *β*-actin served as an internal control.

Furthermore, the apoptosis induction activity on HeLa cells was confirmed by protein expression using Western blotting. Generally, apoptosis is modulated by proapoptotic and antiapoptotic proteins which were used as a target of anticancer therapy. Main two different pathways of apoptosis are mitochondrial or intrinsic and death receptor or extrinsic pathway [31]. In our study, we selected the representative apoptosis-related protein in both pathways, namely, bax and bcl-2 (intrinsic pathway), and caspase-8 and cleaved caspase-8 (extrinsic pathway). We found that bax, a proapoptotic protein, was increased after 48 h of treatment. Meanwhile the expression of bcl-2 (antiproapoptotic protein) and inactive caspase-8 was continuously declined, together with consecutive increasing of active form cleaved caspase-8 (Figure 5). Downregulation of the death suppressor bcl-2 and activation of caspase-8 and bax could inhibit tumor growth through promoting apoptosis. Moreover, intrinsic apoptosis induction is well related with acceleration in bax protein expression which can induce the release of cytochrome c from mitochondria and form with Apaf-1 and caspase-9 called apoptosome complex and it can cleave caspase-9 to active form and then cleave the effector caspases such as caspase-3 and caspase-7 leading to apoptosis. For extrinsic pathway, cleaved caspase-8 is a key molecule that acts as initiator caspase for this process and leads to apoptosis. Meanwhile, cleaved caspase-8 can cleave BID to tBID and tBID fragment then translocate to mitochondria, where it causes cytochrome c release, resulting in apoptosome formation, caspase activation, and cell death [32]. Our results demonstrated that the cytotoxic activity of the ACE extract on HeLa cells caused apoptosis induction in which it may associate with both intrinsic and extrinsic pathways. This hypothesis is supported by previous researches that showed intrinsic and extrinsic activation of* Emilia sonchifolia* extract on colorectal cell line [33] and* Solanum lyratum *extract on leukemia cell line by increased bax and cleaved caspase-8, while decreasing caspase-8 [34].

## 5. Conclusion

The acetone cotyledon extract from VDS fruit showed high cytotoxicity on cervical cancer cells and low toxicity on normal fibroblast cells. This cytotoxic effect caused inhibition in cell growth and the mechanism of this action was apoptosis in HeLa and SiHa cells. Further phytochemistry to obtain a potentially active compound will be undertaken and combination of this extract with chemotherapy agent will be carried out for further study.

## Figures and Tables

**Table 1:** The IC_50_ of crude extracts isolated from VDS fruit type SS.

IC_50_ value for cytotoxicity (*μ*g/mL)					
Cell type	Cell line	Pericarp		Cotyledon	
		Acetone	Methanol	Acetone	Methanol
Cervical cancer	HeLa	17.93 + 0.81	13.40 + 0.71	7.69 + 0.44	11.60 + 0.96
	SiHa	19.00 + 3.37	14.55 + 0.69	9.81 + 1.38	14.54 + 1.10
Normal cell	L929	33.12 ± 0.82	14.10 ± 0.47	34.41 ± 2.05	24.32 ± 1.81

**Table 2:** Selectivity index (SI) of VDS fruit type SS extracts on HeLa and SiHa cells.

Selectivity Index (SI)				
Cell line	Pericarp		Cotyledon	
	Acetone	Methanol	Acetone	Methanol
HeLa	1.85	1.05	4.47^a^	2.1
SiHa	1.74	0.97	3.51^a^	1.67

^a^SI value > 3 is considered to be high selectivity.

## Data Availability

No data were used to support this study.
